# Idiopathic Intracranial Hypertension

**DOI:** 10.34172/aim.33110

**Published:** 2025-03-01

**Authors:** Chun-Chieh Huang, Yu-Fen Wang

**Affiliations:** ^1^Division of Medical Imaging, Department of Radiology, Far Eastern Memorial Hospital, New Taipei City, 220, Taiwan; ^2^Department of Medical Imaging, National Taiwan University Hospital, Taipei City 100, Taiwan

**Figure 1:**
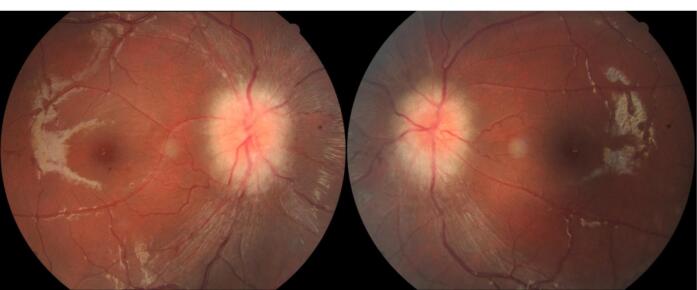
Fundus examination showed markedly increased papilledema in both eyes

 A previously healthy 21-year-old woman with normal body mass index (BMI) and body weight (18.4 kg/m^2^ and 44.3 kg, respectively) experienced progressively worsening, painless blurred vision in both eyes over the past few years, without any systemic symptom. Her vision deteriorated to the point where she could only count fingers (Corrected visual acuity: 20/2000 in both eyes). This was accompanied by a sluggish pupillary light reflex and a failed Ishihara test. Fundus examination revealed significant papilledema in both eyes (Figure 1). However, her cerebrospinal fluid (CSF) and blood tests, including CSF opening pressure, complete blood count (CBC), biochemistry, and immune profile, were normal. Despite receiving steroid pulse therapy twice at another hospital, there was no improvement in her condition.

**Figure 2:**
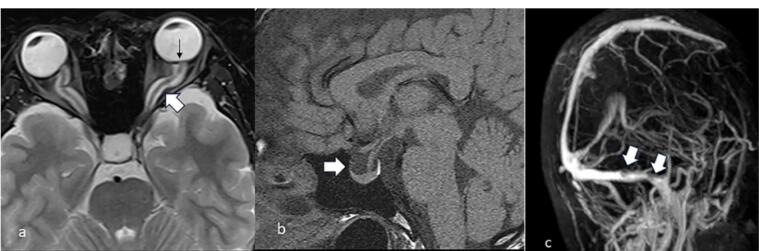
Brain magnetic resonance imaging (MRI) of this 21-year-old woman on T2-weighted image **(a)** showed a bilateral tortuous optic nerve with a prominent surrounding subarachnoid space (white arrow) and flattening of the posterior eye globe (black arrow). On T1-weighted image **(b)**, there is an empty sella (white arrow) and stenosis at the left transverse sinus-sigmoid sinus junction (white arrow) on MR venography **(c)**

 A brain magnetic resonance imaging (MRI) was conducted to rule out potential obstructive structural lesions. The results revealed bilateral tortuous optic nerves with a prominent surrounding subarachnoid space, flattening of the posterior eye globes, an empty sella and stenosis at the left transverse sinus-sigmoid sinus junction (Figure 2a-2c).

**Figure 3:**
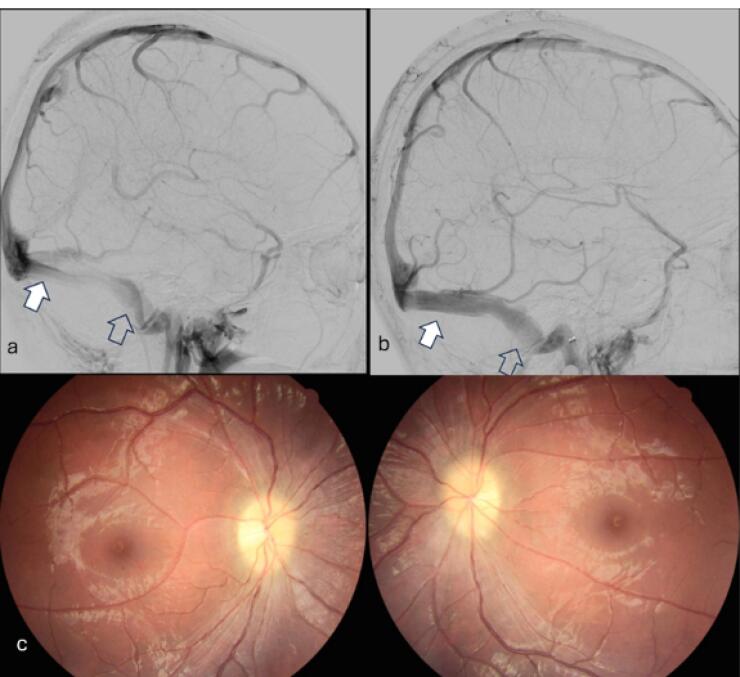
On cerebral angiography **(a)**, contrast inhomogeneity is observed in the stenotic region (between the white and hollow arrows). The measured venous pressure was 74 cm H2O in the pre-stenotic left transverse sinus (white arrow) and 39 cm H2O in the post-stenotic left sigmoid sinus (hollow arrow), revealing a 26 cm H2O pressure gradient. Immediately after balloon dilatation and venous stent placement, contrast homogeneity is observed in the previously stenotic region with no pressure gradient on cerebral angiography **(b)**. One month after venous stenting, fundus examination revealed the absence of papilledema in both eyes **(c)**

 Cerebral venography and manometry revealed stenosis of the left transverse sinus with a trans-stenotic pressure gradient of 35 cmH_2_O (Figure 3a). Balloon dilatation and venous stent placement were performed, reducing the pressure gradient to 0 cmH_2_O (Figure 3b). One month after the procedure, her Corrected Visual Acuity (CVA) improved to 20/200 in both eyes, and fundus examination showed no papilledema (Figure 3c).

 Idiopathic intracranial hypertension (IIH) often presents with symptoms such as headache (75‒94%), pulsatile tinnitus (52%‒60%), photophobia (42%‒73%), and visual loss or blurring (32%).^1,2^ According to the revised diagnostic criteria,^3^ IIH is diagnosed by meeting criteria A to E in the absence of known secondary causes. A ‘probable’ diagnosis of IIH may be considered if only criteria A–D are met. These criteria are defined as follows: (A) Papilledema, (B) Normal neurological examination except for cranial nerve abnormalities, (C) Neuroimaging showing no hydrocephalus, mass, structural lesion, abnormal meningeal enhancement, or venous sinus thrombosis, (D) Normal CSF composition, and (E) Raised lumbar puncture opening pressure (>25 cmH_2_O in lateral decubitus). Additionally, a diagnosis of IIH can be made in patients without papilledema if criteria B–E are satisfied and the patient has a unilateral or bilateral abducens nerve palsy. The diagnosis of IIH can be also ‘suggested’ based on at least three of the following neuroimaging criteria which indicate signs of elevated intracranial pressure: (*i*) empty sella, (*ii*) flattening of the posterior aspect of the globe, (*iii*) distention of the perioptic subarachnoid space with or without a tortuous optic nerve, and (*iv*) transverse venous sinus stenosis, in patients who meet criteria B–E but do not have papilledema or sixth nerve palsy. However, the diagnostic value of abducens nerve palsy as a separate diagnostic criterion has been questioned by Korsbæk et al.^4^ Since empty sella can also occur in healthy individuals and is associated with obesity and other conditions, Beier et al suggest that an empty sella may not be directly associated with IIH. Instead, they propose that the presence of 2 of the 3 MRI signs (posterior globe flattening, optic nerve disc protrusion, and transverse sinus venous stenosis) are the key features for diagnosing IIH.^5^

 The exact cause of IIH remains unknown,^1,2^ with hypotheses suggesting overproduction of CSF, reduced CSF drainage, or increased venous pressure.^2^ Female gender and obesity are recognized risk factors,^1,2,6,7^ but prepubertal thin girls and boys can also have IIH.^3^

 There is no large, randomized trial for IIH treatment.^2^ Weight loss and medication, particularly acetazolamide, which is believed to reduce CSF secretion at the choroid plexus, are the mainstays of treatment.^1,2,6,7^ For patients with mild visual loss due to IIH, there is Class 1 evidence supporting the use of acetazolamide combined with a low-salt diet to improve visual function.^2^

 Surgical options, including CSF diversion via lumboperitoneal/ventriculoperitoneal shunts, optic nerve sheath fenestration, and venous sinus stenting, are reserved for patients who do not respond to conservative treatment or have progressive visual loss.^1,2,7^ While no technique has proven superior, the choice of treatment should depend on the expertise of the hospital.^2^

 This case underscores the diagnostic challenge of IIH in non-obese patients with normal CSF pressure. In this instance, specific MRI signs (posterior globe flattening and transverse sinus stenosis) can aid in diagnosing IIH in patients without typical risk factors.
